# Maximizing adherence and retention for women living with HIV and their infants in Kenya (MOTIVATE! study): study protocol for a randomized controlled trial

**DOI:** 10.1186/s13063-018-2464-3

**Published:** 2018-01-29

**Authors:** Thomas A. Odeny, Maricianah Onono, Kevin Owuor, Anna Helova, Iris Wanga, Elizabeth A. Bukusi, Janet M. Turan, Lisa L. Abuogi

**Affiliations:** 1 Center for Microbiology Research, Kenya Medical Research Institute, Nairobi, Kenya; 2 Department of Health Care Policy and Organization, School of Public Health, University of Alabama at Birmingham, Birmingham, Alabama USA; 3 Department of Pediatrics, University of Colorado, Denver, Colorado USA; 4 Department of Medicine, University of Missouri-Kansas City, Kansas City, Missouri USA

**Keywords:** Text messages, SMS, Community mentor mothers, PMTCT, HIV, Option B+

## Abstract

**Background:**

Successful completion and retention throughout the multi-step cascade of prevention of mother-to-child HIV transmission (PMTCT) remains difficult to achieve. The Mother and Infant Visit Adherence and Treatment Engagement study aims to evaluate the effect of mobile text messaging, community-based mentor mothers (cMMs), or both on increasing antiretroviral therapy (ART) adherence, retention in HIV care, maternal viral load suppression, and mother-to-child HIV transmission for mother-infant pairs receiving lifelong ART.

**Methods/design:**

This study is a cluster randomized, 2 × 2 factorial, controlled trial. The trial will be undertaken in the western Kenyan counties of Migori, Kisumu, and Homa Bay. Study sites will be randomized into one of four groups: six sites will implement both text messaging and cMM, six sites will implement cMM only, six sites will implement text messaging only, and six sites will implement the existing standard of care. The primary analysis will be based on the intention-to-treat principle and will compare maternal ART adherence and maternal retention in care.

**Discussion:**

This study will determine the impact of long-term (up to 12 months postpartum) text messaging and cMMs on retention in and adherence to ART among pregnant and breastfeeding women living with HIV in Kenya. It will address key gaps in our understanding of what interventions may successfully promote long-term retention in the PMTCT cascade of care.

**Trial registration:**

ClinicalTrials.gov, NCT02491177. Registered on 11 March 2015.

**Electronic supplementary material:**

The online version of this article (10.1186/s13063-018-2464-3) contains supplementary material, which is available to authorized users.

## Background

There has been a remarkable increase in the proportion of HIV-infected pregnant women receiving antiretroviral therapy (ART) globally from 37% in 2009 to 77% in 2015 [1]. As a result, an estimated 1.1 million new HIV infections among children have been averted, and AIDS-related deaths among HIV-infected pregnant women have declined by 45% [1]. In 2015, the World Health Organization (WHO) recommended providing lifelong ART to all HIV-infected pregnant and breastfeeding women, regardless of CD4 cell count or clinical stage [2]. This guideline, termed “Option B+,” is expected to lead to even greater declines in both the number of new HIV infections and AIDS-related maternal deaths.

The Option B+ approach was designed in part to reduce barriers to ART initiation for pregnant and breastfeeding women, such as the need for CD4 testing to determine treatment eligibility and complex treatment regimens. However, successful completion and retention throughout the multi-step prevention of mother-to-child HIV transmission (PMTCT) cascade of care remains difficult to achieve, threatening the great potential of this strategy. The Option B+ approach may pave the way to achieving virologic suppression and potentially eliminating mother-to-child HIV transmission, yet the high potential for attrition and disengagement from care increases the risk of morbidity and mortality for both mother and child.

The challenges of achieving success in the PMTCT cascade are perhaps best highlighted in Malawi’s pioneering Option B+ program. In this program, pregnant women who initiated ART for PMTCT had a fivefold higher risk of failure to return to clinic compared to those who started ART for their own health [3]. Retention in PMTCT in this program has varied between 65 and 87% [3–6]. A similar picture of imperfect engagement in care is observed across other sub-Saharan African Option B+ programs: 88% retained at 6 months in Ethiopia [7], 83% retained at 6 months in Zimbabwe [8], 66% retained at 1 year in Ghana [9], and 66% completing postnatal follow-up in Nigeria [10]. Further, among women who initiate ART and are later transferred from PMTCT clinics in the postpartum period to general adult ART clinics, about 25% disengage from care after referral [11]. A systematic review and meta-analysis of 44 studies from 15 sub-Saharan African countries reported significant drop-offs along the cascade of PMTCT care for mother-baby pairs: 70% of HIV-positive pregnant women received antiretroviral prophylaxis, 64% of HIV-exposed infants were tested for HIV using DNA polymerase chain reaction (PCR) at 6 weeks after birth, and 55% were tested between 1 year and 18 months of age [12]. These high rates of attrition reflect the precarious steps along the cascade of care and treatment for pregnant and breastfeeding women living with HIV and their infants. For Option B+ to succeed, it is necessary to identify optimal models of care that support maternal adherence and maternal and infant retention through the care continuum. There is an urgent need to investigate efficacious, cost-effective, and sustainable interventions to improve maternal and infant retention and adherence to lifelong ART in the setting of Option B+.

Existing research documenting evidence-based interventions to promote retention in this population is limited, as reflected by the recently published systematic review of interventions to improve postpartum retention of women in PMTCT and ART care [13]. However, there is growing evidence that community-based interventions that empower people living with HIV and mobile phone interventions may be powerful approaches to improve retention along the PMTCT cascade [14–16]. The Joint United Nations Programme on HIV/AIDS (UNAIDS) highlights the need for community and, in particular, female empowerment [17]. The Government of Kenya supports the principle that women living with HIV must be at the center of the response to the epidemic [18, 19] and also supports a facility-based mentor mother approach as outlined in the Kenya Mentor Mother Program launched in 2012 [20]. Separately, mobile phone interventions have shown promise for supporting service provision and retention of and adherence to HIV programs [21, 22]. In one randomized trial in Kenya, text messaging significantly improved maternal retention in the early postpartum period and increased rates of testing to facilitate early infant diagnosis of HIV [23, 24].

Although mentor mother approaches may enhance adherence and retention in care, little is known about the benefit of supplementing this strategy with mobile phone interventions and implementing the strategy at the community level. Using a 2 × 2 factorial cluster randomized trial, the Mother and Infant Visit Adherence and Treatment Engagement (MOTIVATE!) study aims to evaluate the effect of mobile text messaging, community-based mentor mothers (cMMs), or both on increasing ART adherence, retention in HIV care, maternal viral load suppression, and mother-to-child HIV transmission for mother-infant pairs receiving lifelong ART (Option B+).

## Methods/design

### Study setting and sites

The focus of this work is in Migori, Kisumu, and Homa Bay counties in Kenya. With 14.3% (Migori), 19.9% (Kisumu), and 26% (Homa Bay) of adults 15–49 years of age testing HIV-positive in 2015, this region has the highest HIV prevalence in Kenya [25]. Kenya officially adopted Option B+ for pregnant and breastfeeding women in 2014, with rollout thereafter. This setting has several characteristics that make it a priority area for research on the implementation of Option B+ among HIV-positive pregnant women and their families, including (1) high HIV prevalence among pregnant women (20%), (2) high rates of MTCT of HIV (10%), and (3) low retention for women who test HIV-positive at antenatal care (ANC) clinics [26, 27]. This study will be conducted at 24 low-resource primary health care facilities in the selected counties. All study facilities provide PMTCT services including Option B+ integrated into the ANC clinic.

### Study design

**Fig. 1:**
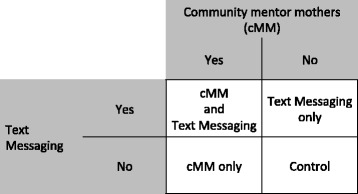
2 × 2 factorial design. Using a 2 × 2 factorial design, this study will evaluate the effect of community-based mentor mothers (cMMs), text messaging, or both on service utilization and maternal and child health outcome indicators

This study is a cluster randomized, factorial, controlled trial. Using a 2 × 2 factorial design, we will evaluate the effect of cMMs, text messaging, or both on service utilization and maternal and child health outcome indicators. Study sites will be randomized into one of four groups: six sites will implement both interventions, six sites will implement the cMM intervention only, six sites will implement the mobile phone text message intervention only, and six sites will implement the existing standard of care only (Fig. 1). Randomization will be stratified by geographic region such that each unique geographic region will have at least one site in each study arm. The study employs a unique participatory community randomization approach. Site representatives and key stakeholders will be invited to a centralized randomization meeting in which a representative from each site chooses a sealed opaque envelope from a jar; the envelope reveals their site’s randomization arm.

The interventions will be added to fully integrated high-quality HIV and antenatal, maternal, neonatal, and child health (ANC/MNCH) services already offered at these sites. These services will be enhanced with regular Continuing Medical Education seminars and customer care/stigma-reduction training for health workers.

### Participants

We aim to enroll 1336 HIV-infected pregnant women identified in the ANC clinics at study sites. We are including HIV-infected pregnant women who are 18 years or older, have access to a mobile phone (and have disclosed their HIV status to any person sharing the phone), and are willing to have home visits (or meetings with a cMM in an alternate location). We are excluding non-pregnant women, those with unknown HIV status, or those uninfected at the time of the first ANC visit. Approximately 334 women will be enrolled in each of the four study arms. HIV-positive pregnant women attending ANC clinics at one of the study sites will be recruited by a study nurse at presentation to clinic or referral from the community. Informed consent will be obtained for all participants. Women will be sequentially recruited until the sample size has been attained.

### Interventions

Our interventions build on comprehensive integrated HIV and ANC/MNCH services at the clinic level, adding evidence-based approaches in the community and at the individual level to support adherence to and retention in the setting of Option B+. These include (1) cMMs who conduct home visits and other community outreach activities to support PMTCT services and (2) theory-based mobile phone text messages with bi-directional communication to relay health messages and help retain women, men, and infants in existing clinical services.

#### cMMs

cMMs are women living with HIV with recent pregnancy experience who support uptake and retention of PMTCT services in their communities. Unlike traditional facility-based mentor mother programs, in this study, cMMs will be based in the community and will conduct home visits for HIV-positive women (together with their male partners if desired) in their respective communities in order to assist with safe disclosure, support safe infant feeding, promote safer sex and family planning, encourage early infant testing and follow-up, and promote ART adherence and return for HIV care visits.

Criteria for selecting cMMs are modeled on Kenya Mentor Mother Program Guidelines [20]. These include being an HIV-positive mother, having recent PMTCT experience (6 months–2 years), attaining a minimum of Standard 8 education, having disclosed HIV status to at least one person within her household, living within the local community, and demonstrating good ART adherence for at least 6 months. cMMs will participate in 2 weeks of initial training that covers basic medical knowledge about HIV infection and ART, behaviors that help prevent mother-to-child transmission, safer feeding options for infants, counseling methods that can help women to safely disclose their status, strategies for negotiating safer sexual practices, and nutritional guidelines for women living with HIV as outlined in the Kenya Mentor Mother National Guidelines [20]. This training will include stigma-reduction and sensitivity training modules.

For this study, the home locations provided by the enrolled pregnant women will be grouped into zones. Each zone will comprise approximately 10 villages and will correspond to the geographic sub-locations in the community unit. Each zone will be given a unique identifying number and will have two to three resident cMMs who will also be identified by that number. The research staff will obtain informed consent from the HIV-positive woman for home visits by the cMM. Women who do not wish to be visited at home but who accept meetings with cMMs will be given the option to meet at an alternate location. Each cMM will be initially assigned 25–30 women, whom she will follow through pregnancy and 12 months postpartum. The cMM visit schedule is designed to coordinate with ANC visits, maternal clinic visits, and infant HIV testing and immunization schedules to maximize retention in PMTCT and other essential health services for women and children as outlined in the national community maternal newborn health (CMNH) guidelines for Kenya. Specifically, cMMs will conduct at least two prenatal visits 4 weeks apart, one visit on the day of delivery (day 0) and additional postnatal visits on days 2 and 7, weeks 6, 10, and 14, and at months 6, 9, and 12. During home visits, cMMs will provide HIV and other health education, support HIV disclosure and ART adherence, and facilitate retention in care. To assist with their duties, cMMs will each be given a bicycle, identifying study T-shirts, a mobile phone, a CMNH counseling chart, basic supplies, and an initial basic monthly allowance of about $117 USD. A full-time study nurse will perform day-to-day supervision of cMM activities in each study zone.

#### Mobile phone text messaging

Previously tested text messages sent antenatally through 6 weeks postpartum in this setting were adopted from Odeny et al. [23, 24]. We conducted additional focus group discussions to refine the content, optimum frequency, and timing of the additional text messages to be used in this study, focusing on ART adherence and retention in care for Option B+ [28]. Based on focus group input, HIV will not be explicitly mentioned in text messages. However, medication adherence will be emphasized along with reminders for return clinic visits and general health messages. Participating women will receive text messages at their preferred frequency and in their preferred language (from a choice of English, Kiswahili, or Dholuo). Women who share phones will be enrolled only if they have disclosed their HIV status to the person with whom the phone is shared. From our previous work with mobile technologies for HIV prevention and treatment, we have developed an automated text messaging software that allows customization of messages based on an individual recipient’s preferred language and preferred time for receiving messages. Further individual tailoring allows options for messages to include a participant’s name as well as her infant’s name and sex after delivery. Participants’ phone numbers and text preferences will be recorded by sending a text message from their mobile phone to our automated software using a pre-defined syntax. The syntax will include their unique study identification number, date of the last normal menstrual period, preferred language, a preferred time for receiving texts, and an option to provide a preferred name. Participants’ mobile phones will be loaded with 20 Kenya Shillings (approximately $0.20 USD) worth of airtime to cover the cost of this registration message. After registration, our automated study software will send out free messages weekly, at the preferred time of day and in the language specified by the participant. Weekly message content is intended to coincide with PMTCT care and maternal child health schedules and is sent through 1 year postpartum. A bi-directional communication component will supplement the automated text messages to provide additional support and education for women. At any time during the study, participants will be allowed to call or send a text to the study coordinator, a nurse trained and experienced in PMTCT and maternal and child health. Participants will be able to request to talk with the study coordinator by sending a free “call back” message. Whenever a participant requests to be called back, our study software will immediately send back a confirmation response and simultaneously send a text message to alert the study coordinator. The coordinator will then call the patient and record details of their conversation.

### Study procedures

On-the-job training to strengthen the delivery of fully integrated ANC/MNCH/HIV clinical services will be conducted. Refresher trainings for health care providers at study sites will cover the components of high-quality ANC, PMTCT, and HIV treatment for pregnant women; early infant diagnosis; care and treatment for HIV-exposed infants and HIV-infected children; and postpartum family planning.

Study interventions will begin during pregnancy when the participant is enrolled and continue up to12 months postpartum. Blood sampling and laboratory testing required for primary and secondary study outcomes, including HIV testing for pregnant women and exposed infants, maternal CD4 counts, and viral loads, will be conducted as part of routine care per Kenya National ART guidelines by health facility staff.

We will also establish a Community Advisory Group (CAG) composed of local health workers, community leaders, representatives of community groups, and persons living with HIV in the study communities. This group will be invited to participate in a multi-day workshop to discuss the study plans, refine the content of community outreach messages, and discuss the cMM intervention; as well as in regular meetings throughout the study period. We will work with the CAG to develop methods for introducing the study, obtaining local support, and keeping communities informed about study progress and results.

### Data collection and measures

Standardized HIV clinic visit forms and clinic registers developed by the Kenyan Ministry of Health (MOH) are used at all patient clinic visits. For this study, a standardized electronic data abstraction form with built-in quality controls has been developed to capture demographic and clinical information from these clinic and patient records. Data will be collected by trained research staff from source documents on a weekly or bi-weekly basis. All participant visits since the last data abstraction will be entered into the electronic form. Research staff will support accurate and thorough documentation by health care providers during routine site visits. Data collection on study participants will continue for a minimum of 12 months postpartum or until study end.

Primary outcome measures include adherence to ART and retention in care at 12 months postpartum. ART adherence will be measured via self-report routinely collected in the woman’s medical record and viral load. Patient adherence is classified by MOH routine data collection tools as good (>95%), fair (85–94%), or poor (<85%) assessed from the last clinic visit, with clear definitions for each of these categories. Viral load measurements will be conducted as per Kenya National ART guidelines after 6 months of ART for those newly initiating (or at time of pregnancy identification for those already on ART). Those with viral load < 1000 copies/mL will be considered virally suppressed. Those with viral load > 1000 copies/mL will be evaluated for evidence of treatment failure or poor adherence. Our primary outcome for retention in care will be obtained from the women’s clinic visit history and is defined as the proportion of women who have an HIV care visit within 90 days at 12 months after delivery. Data will be shared in agreement with funder (National Institutes of Health, NIH) data sharing policies. Results will be disseminated to local stakeholders (including participants) through local presentations, regional/national/international conferences, and publications.

### Power and sample size estimation

We have powered this study based on differences in proportions in a cluster randomized trial for our key outcomes of maternal ART adherence (percentage of women who report at least 85% adherence) and maternal retention in care (proportion of women with visit within 90 days) at 12 months postpartum. Our sample size and power estimates are based on both clinical and logistic factors. Current estimates on required adherence to non-nucleoside-based regimens are estimated to be 75–80% [29–31]. Additionally, current standard HIV clinic forms in use in Kenya define good adherence as > 95% adherence, fair adherence as 85–94% adherence, and poor adherence as < 85% adherence based on number of missed doses. Power calculations were made based on estimated differences in these rates in the intervention versus the control arms of the study, using appropriate methods for clustered data [32]. We estimated a baseline ART adherence rate of 55% based on current rates found in the literature for sub-Saharan Africa [33] and a baseline retention in care rate of 48% based on data from our recent trial of service integration conducted in the same region in Kenya [26]. We powered our study to be able to detect a 25% absolute increase in ART adherence due to each intervention individually. Given that the actual intracluster correlation coefficients (ICCs) are not known for these outcomes and are not easily specified, we calculated the number of communities needed for ICCs ranging from 0.06–0.12 for 80% power with a two-sided alpha of 0.05, for a fixed average cluster size (Table 1). To achieve 80% power for detecting a significant difference in the proportion who are at least 85% adherent at 12 months, from 55% in the control arm versus 80% in the intervention arm (two-sided *p* < 0.05), with a conservative (high) ICC of 0.12 and an average of 70 women sampled per community (an estimated minimum of 50 and maximum of 130 women per cluster), we would need 18 communities, 4.5 in each arm of the trial. For retention in care, to achieve 80% power to detect a significant difference in the proportion who have an HIV care visit within 90 days of the 12 months postpartum date, from an estimated 48% in the control arm versus 73% in the intervention arm (two-sided *p* < 0.05), with a conservative (high) ICC of 0.12 and an average of 70 women sampled per community (50—130 women per cluster), we would need 20 communities, 5 in each arm of the trial. This sample size will power us to evaluate both of our primary outcomes. It is conservative due to the high ICC estimate and thus should also be sufficient if we experience loss to follow-up. This will result in a target sample size of 1336 women (334 women per arm).

**Table 1:** Sample size calculations with different levels of intracluster correlation coefficient (ICC)^a^

Adherence (proportion of women at least 85% adherent at 12 months postpartum)					
ICC	Control	Difference	Intervention	Number of sites	Total sample
0.06	55%	25%	80%	10	680
0.07	55%	25%	80%	12	772
0.08	55%	25%	80%	12	866
0.09	55%	25%	80%	14	958
0.10	55%	25%	80%	16	1050
0.11	55%	25%	80%	16	1144
0.12	55%	25%	80%	18	1236
Retention in care (proportion of women with an HIV visit within last 90 days at 12 months postpartum)					
ICC	Control	Difference	Intervention	Number of sites	Total sample
0.06	48%	25%	73%	10	734
0.07	48%	25%	73%	12	834
0.08	48%	25%	73%	14	934
0.09	48%	25%	73%	14	1036
0.10	48%	25%	73%	16	1136
0.11	48%	25%	73%	18	1236
0.12	48%	25%	73%	20	1336

^a^Estimates adjusted for unequal cluster sizes, assuming an average cluster size of 70

### Primary analyses

Preliminary analyses will summarize characteristics of the intervention and control women and assess frequency distributions of variables to identify outlying or unusual values. Initial analyses of our primary outcomes of maternal ART adherence and maternal retention in care (dichotomous variables) will be based on comparisons of proportions between intervention and control groups using cluster-adjusted chi square tests. Although there are only 24 clusters, each mean or percentage will be based on a relatively large number of women, and the normal approximation to the binomial distribution can be used. These tests will be evaluated based on a two-sided alternative hypothesis with a 5% type I error rate. Given the clustered study design, generalized estimating equation (GEE) models will be used to test for differences of interest in univariate and multi-variable models, adjusted for relevant covariates (such as time on ART), using logistic links for these binary outcomes. Secondary outcomes, such as time to infant testing uptake, maternal viral load suppression, and changes in maternal CD4 counts, will be analyzed using similar methods, taking into account the nature of the dependent variable (continuous, dichotomous, or time to event). Results will be summarized as odds ratios for models based on binary responses (e.g., enrollment in HIV care), hazard ratios for time-to-event outcomes (e.g., time to infant HIV test), and mean changes for continuous responses (e.g., maternal CD4 count); respective 95% confidence intervals will also be reported based on the robust variance estimates from the GEE models.

### Secondary analyses

Past research with this population suggests that, in spite of our best efforts, we will be unable to locate some individuals for follow-up, perhaps as many as 20%. We will gauge the impact on our study in two ways. First, we will perform multiple imputations under the missing-at-random (MAR) assumption. Our imputation model will include the rich array of baseline covariates collected; in that context, MAR would mean that women we locate are representative of women with the same observed characteristics whom we cannot locate. This assumption is superior to a range of other ad hoc methods; however, the MAR assumption is untestable [34, 35]. For that reason, we will also examine the sensitivity of our findings to violations of the MAR assumption, the so-called non-ignorable non-response. We will also consider inverse probability of censoring weighting (IPCW), which is regression analysis inversely weighted by the probability of participation; it is determined using a logistic regression model for probability of participation given baseline or previous history of covariates and outcomes. IPCW inflates the impact of underrepresented subjects, so we can observe associations that would have been observed if all subjects had stayed in the study to completion.

## Discussion

This study will determine the impact of long-term (up to 12 months postpartum) text messaging and cMMs on retention in and adherence to ART among pregnant and breastfeeding women living with HIV in Kenya. It will address key gaps in our understanding of what interventions may successfully promote long-term retention in the PMTCT cascade of care. While support among women living with HIV appears to be high for Option B+, high rates of “non-starters” (women who default after first visit) and losses of up to a third of women on ART are concerning [36–39]. The full benefits of Option B+ can only be realized if women are adherent to ART during pregnancy, breastfeeding, and throughout life.

Community-based strategies have the potential to enhance adherence and retention. Most countries in sub-Saharan Africa utilize community or lay health workers to support HIV services. However these workers may or may not be people living with HIV (PLHIV), and the majority are facility-based. While this has its benefits, the WHO and UNAIDS strongly recommend involvement of PLHIV in their own care [40]. Models utilizing PLHIV to support HIV services, such as peer navigators or the Mother-to-Mother program specifically for PMTCT, have shown some benefit but require rigorous evaluation. Our study will be able to determine the separate and combined effectiveness of cMMs and text messages specifically for women on Option B+.

In Kenya, a country of 43 million people, there are currently 37.8 million active mobile phone subscriptions, representing about 88% of the population [41]. While access to a mobile phone is significantly associated with postpartum retention in PMTCT [42–44], there are few studies evaluating the long-term impact of mobile phone technologies on improving long-term retention and adherence in the context of Option B+. One cluster randomized trial of text messages to support PMTCT care in Kenya did not demonstrate increased uptake of PMTCT services such as ART uptake, facility delivery, or infant testing, but it did not study the effects long term [45]. Another recent randomized trial in Kenya used behavior-based text messaging to improve uptake of early infant diagnosis and postnatal retention [23]. Our study will build on the results of this successful trial by using theory-based behavior text messaging to support long-term adherence and retention in the context of Option B+.

The use of the 2 × 2 factorial design will allow us to examine the individual effects of each intervention, as well as the potential combined effects when both interventions are introduced in the same community. Although the interventions we will test alone and in combination have been used in sub-Saharan Africa and are evidence-based, we will include some novel aspects that our prior research suggests will make these interventions even more effective. These include basing mentor mothers in the community rather than at the health facility and focusing their activities on HIV prevention and treatment behaviors as well as support for adherence and retention among women and children. Similarly, our mobile phone intervention will extend beyond the short-term interventions that have previously been tested for PMTCT to continue for a minimum of 12 months and will include unique theory-based content as well as bi-directional communication to support long-term adherence and retention.

Our study has a number of limitations. First, it is being conducted in an environment where study sites are implementing other interventions simultaneously. This might attenuate the effect of our intervention. Second, policy and service changes are constantly occurring within the PMTCT field which might interact with our intervention in unknown ways. However, we feel that operating in an environment with other simultaneous interventions and changing policies reflects “real-world” implementation of our interventions and therefore enhances external validity. Finally, our study relies on medical records and a single-item self-report of ART adherence. This method carries biases that might result in inaccurate measurement of medication adherence. To address this limitation, we will also measure HIV viral load, which is a biological measure that is less prone to bias.

In summary, this trial uses novel interventions and research methods to study an issue in a setting where lifelong ART for women living with HIV (Option B+) has recently been rolled out countrywide and has not been studied. Our study will provide important guidance for similar countries in sub-Saharan Africa by identifying effective and scalable interventions and combinations of interventions that can reduce barriers and increase facilitators of optimal ART adherence and retention in care for pregnant and breastfeeding women on ART. The aims are to reach the elimination of mother-to-child transmission of HIV and significantly improve maternal health.

## Trial status

**Fig. 2:**
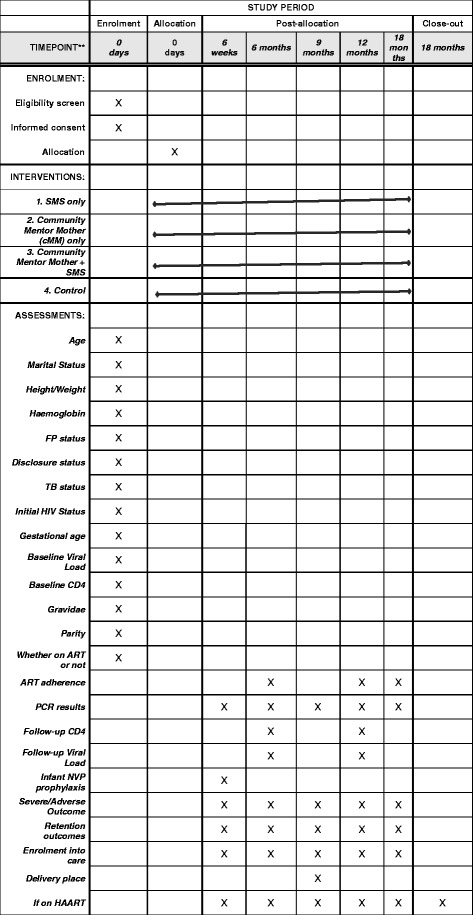
Standard Protocol Items: Recommendations for Interventional Trials Statement (SPIRIT) figure for the MOTIVATE! study. Schedule of enrollment, intervention and assessments

**Fig. 3:**
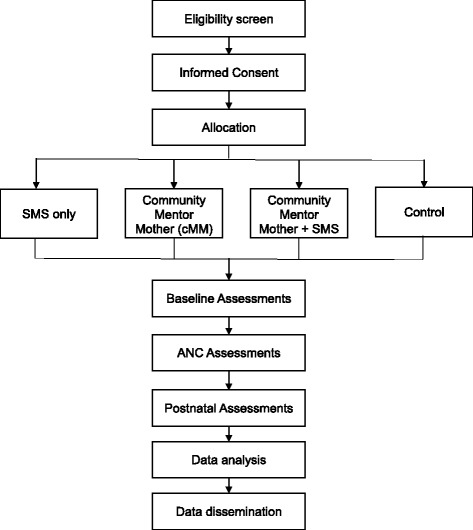
Flow Diagram for the MOTIVATE! study

The MOTIVATE! study is currently in month 44 of 60 planned months of recruitment and data collection. A populated Standard Protocol Items: Recommendations for Interventional Trials (SPIRIT) checklist and a figure describing the study protocol are included as Additional file 1 and Fig. 2. The flow diagram is presented in Fig. 3.

## Additional file

Additional file 1: Standard Protocol Item: Recommendation for Interventional Trials (SPIRIT) checklist for the MOTIVATE! study. (DOC 123 kb)

## Abbreviations

ART: Antiretroviral therapy
cMM: Community-based mentor mother
MOTIVATE!: Mother and Infant Visit Adherence and Treatment Engagement (study)
Option B+: Lifelong ART for women living with HIV
PMTCT: Maternal and child health prevention of mother-to-child HIV transmission
SMS: Short message service
